# Association of uPA and PAI-1 tumor levels and 4G/5G variants of *PAI-1* gene with disease outcome in luminal HER2-negative node-negative breast cancer patients treated with adjuvant endocrine therapy

**DOI:** 10.1186/s12885-018-5255-z

**Published:** 2019-01-15

**Authors:** Marko Jevrić, Ivana Z. Matić, Ana Krivokuća, Marija Đorđić Crnogorac, Irina Besu, Ana Damjanović, Mirjana Branković-Magić, Zorka Milovanović, Dušica Gavrilović, Snezana Susnjar, Darija Kisić Tepavčević, Tatjana Stanojković

**Affiliations:** 1 Institute of Oncology and Radiology of Serbia, Pasterova 14, Belgrade, 11000 Serbia; 2 Institute of Epidemiology, Faculty of Medicine, University of Belgrade, Višegradska 26, Belgrade, 11000 Serbia

**Keywords:** uPA, PAI-1, *PAI-1* –675 4G/5G polymorphism, Prognostic, Luminal/HER2-negative, node-negative breast cancer, Adjuvant endocrine therapy

## Abstract

**Background:**

The aim of this study was to evaluate the prognostic potential of urokinase-type plasminogen activator (uPA) and plasminogen activator inhibitor type 1 (PAI-1) tumor tissue levels and examine the association between these biomarkers and classical prognostic factors in early node-negative luminal breast cancer patients. The clinical value of 4G/5G variants of *PAI-1* gene was evaluated.

**Patients and methods:**

This study involved 81 node-negative, estrogen receptor-positive and/or progesterone receptor-positive and human epidermal growth factor receptor 2-negative operable breast cancer patients who underwent radical surgical resection and received adjuvant endocrine therapy. Determination of uPA and PAI-1 concentrations in the breast cancer tissue extracts was performed using FEMTELLE® uPA/PAI-1 ELISA. An insertion (5G)/deletion (4G) polymorphism at position − 675 of the *PAI*-1 gene was detected by PCR-RFLP analysis.

**Results:**

Our research showed that patients with uPA tumor tissue levels higher than 3 ng/mg of protein had significantly reduced disease-free survival (DFS) and overall survival (OS) when compared to patients with uPA tumor tissue levels lower or equal to 3 ng/mg of protein. Patients with PAI-1 tumor tissue levels higher than 14 ng/mg of protein had significantly decreased OS in comparison with patients with PAI-1 tumor tissue levels lower or equal to 14 ng/mg of protein. ROC analysis confirmed the uPA and PAI-1 discriminative potential for the presence/absence of relevant events in these patients and resulted in higher cut-off values (5.65 ng/mg of protein for uPA and 27.10 ng/mg of protein for PAI-1) than standard reference cut-off values for both biomarkers. The prognostic importance of uPA and PAI-1 ROC cut-off values was confirmed by the impact of uPA higher than 5.65 ng/mg of protein and PAI-1 higher than 27.10 ng/mg of protein on poorer DFS, OS and event-free survival (EFS).

We observed that patients with dominant allele in *PAI-1* genotype (heterozygote and dominant homozygote, − 675 4G/5G and − 675 5G/5G) had significantly increased DFS, OS and EFS when compared with patients with recessive homozygote genotype (− 675 4G/4G).

**Conclusion:**

Our study indicates that uPA and PAI-1 tumor tissue levels and 4G/5G variants of *PAI-1* gene might be of prognostic significance in early node-negative luminal HER2-negative breast cancer patients treated with adjuvant endocrine therapy.

## Background

Urokinase-type plasminogen activator (uPA) and its inhibitor, plasminogen activator inhibitor type 1 (PAI-1), play essential roles in tumor invasion and metastasis, being involved in degradation of the tumor stroma and basement membrane [1]. Increased levels of uPA and PAI-1 are present in breast carcinomas compared with benign lesions or normal breast tissue [2]. Duffy and colleagues were the first to report that high activity of primary tumor uPA is associated with poor survival in breast cancer patients [3], which was confirmed later [4]. Several other authors also demonstrated the independent prognostic value of uPA and PAI-1 in breast cancer patients [5–10]. Standard reference cut-off values were set, and elevated levels of both markers were associated with poor prognosis [11]. The clinical relevance of uPA and PAI-1 tumor tissue levels in providing risk group discrimination is the greatest when they are used in combination compared to either factor alone (e.g. both low vs**.** either or both high). [12]. Node-negative patients with low uPA and PAI-1 tumor levels have an excellent prognosis with a 5-year disease-free survival (DFS) exceeding 90%, even without adjuvant systemic therapy [13]. The predictive value of uPA and PAI-1 tumor tissue levels in response to adjuvant chemotherapy was also investigated and confirmed [8, 14–16]. The combined ability of uPA and PAI-1 to predict both outcome and response/resistance to specific therapies should further lead to individualized management of patients with breast cancer, thus helping clinicians to predict treatment efficacy [17].

The effects of uPA are neutralized by plasminogen activator inhibitors 1 and 2 (PAI-1 and 2), produced by stromal cells surrounding the tumor cells [18]. By forming a stable complex with active uPA, PAI-1 determines a negative feedback control with consequent inhibition of plasmin formation [19]. Previous reports have suggested that the insertion (5G)/deletion (4G) polymorphism at position − 675 of the *PAI- 1* gene could influence the amount of PAI-1 synthesis owing to its location in the promoter region in the gene and the effect that it might have on the transcription [20, 21]. These reports showed that the presence of 4G/4G homozygotes enhances transcription to increase plasma PAI-1 levels whereas 5G/5G homozygotes are associated with lower levels of the inhibitor.

The objective of this retrospective analysis was to evaluate the prognostic significance of uPA and PAI-1 breast cancer levels in estrogen receptor (ER)-positive and/or progesterone receptor (PR)-positive and HER2-negative node-negative early breast cancer patients who were treated with adjuvant endocrine therapy. In addition, the association between these biomarkers and classical prognostic factors was examined. The clinical significance of − 675 4G/5G variants of *PAI-1* gene in this group of patients was evaluated as well.

## Patients and methods

### Patients

This study involved 81 patients with operable breast cancer, who underwent surgical resection at the Institute of Oncology and Radiology in Belgrade between 2010 and 2012. Breast cancer specimens obtained during surgical resection were frozen and deposited in the institutional tumor bank. All patients signed Informed consent before surgical treatment in which they consented with the storage of the rest of their tissue samples in the Tumor bank of the Institute. They agreed that samples can be used for further research. All patients had histologically confirmed invasive hormone receptor (HR)-positive/HER2-negative breast cancer and all of them were clinically node-negative, majority of whom were pathologically approved to be node negative. At the time of primary therapy, none of the patients had evidence of distant metastases. Median age of the patients at the time of primary surgery was 66 years (range, 36–82 years). All patients had been treated with loco-regional breast cancer therapy consisting of either modified radical mastectomy (*n* = 34), or breast-conserving surgery and postoperative radiation therapy (*n* = 47).

Adjuvant treatment was performed according to the current guidelines for the diagnosis and the treatment of breast cancer [22]. All patients, except two, were postmenopausal and received endocrine therapy with either selective estrogen receptor modulator tamoxifen, or aromatase inhibitors anastrozol or letrozol. Those two premenopausal patients were treated with the luteinizing hormone-releasing hormone agonists in combination with tamoxifen. None of them received adjuvant chemotherapy.

Classical prognostic factors (menopausal status, pathological tumor size, type and grade of tumor, and nodal status) were determined by clinical and pathological examination of the tumor tissue. Histological type was determined according to the International Union Against Cancer-World Health Organization criteria, and the grade of malignancy was scored according to the Scarff-Bloom-Richardson grading system modified by Elston and Ellis [23].

### Study objective

Prognostic significance of tumor size and grade, ER expression, Ki67, uPA and PAI-1 tumor tissue levels and − 675 4G/5G variants of *PAI-1* gene was estimated by their influence on disease outcomes, defined as follows: a) disease-free survival (DFS): time from radical breast surgery to loco-regional recurrence and/or distant metastases and/or contralateral breast cancer; b) overall survival (OS): time from breast surgery to death from any cause; c) event-free survival (EFS): time from breast surgery to loco-regional recurrence and/or distant metastases and/or contralateral breast cancer and/or non-breast primary cancer or death from any cause.

### Immunohistochemistry (IHC)

Expression levels of ER, PR, HER2 and Ki67 were determined by IHC staining of formalin-fixed paraffin-embedded tumor tissue sections. Cut-off values for positive hormone receptors’ status (Allred score) for both ER and PR were 3–8 [24]. Antibodies used for IHC staining were: anti-human ERα (clone SP1, 1:200 dilution; LabVision), anti-human PR (clone PgR 636, 1:500 dilution; Dako), and anti-human HER2 (clone CB11, 1:800 dilution; Novocastra). Negative HER2 status was defined as IHC 0 and IHC 1+ and IHC 2+/CISH (chromogen in situ hybridization) - negative tumors [25]. Ki67 was determined using LabVision monoclonal antibody (clone SP6, dilution 1:200). For visualization ULTRA vision detection system was used (RTU, ready for use, Lab Vision). The cut off value for the low Ki67 proliferative index was Ki67 less than 15% of breast cancer cells.

### Measurement of uPA and PAI-1 concentrations by ELISA

Tissue protein extracts were prepared by homogenization of frozen breast tumor tissue samples in TBS (Tris Buffered Saline) with Ultra Turrax. Detergent Triton-X 100 was added to tissue homogenates at a final concentration of 1% and the samples were incubated on a shaker for 16 h at 4 °C. After incubation, the tissue extracts were centrifuged at 16,000 g for 1 h at 4 °C. The protein extracts (supernatants) were aliquoted and stored at − 20 °C before analyses. The concentrations of total proteins of the tumor tissue extracts were measured by Pierce™ BCA Protein Assay Kit (Thermo Scientific, catalog number 23227). Determination of uPA and PAI-1 concentrations in the breast tumor tissue extracts was performed using FEMTELLE® uPA/PAI-1 ELISA, according to manufacturer instructions (Sekisui Diagnostics, LLC, Stamford, USA, ref. 899). In brief, the diluted samples of tumor tissue extracts, control and uPA or PAI-1 standards were added to coated wells. The plates were incubated overnight at 8 °C. After washing, detection antibodies were added to the wells and plates were incubated for 1 h at room temperature. Subsequently, the plates were washed and uPA or PAI-1 enzyme conjugate was added to the wells. After 1 h incubation, the plates were washed and TMB substrate solution was added to each well. The plates were incubated for 20 min and the enzymatic reaction was stopped by adding H_2_SO_4_. The absorbance was measured at a wavelength of 450 nm using Multiskan EX Thermo Labsystems plate reader. All samples were analyzed in duplicate. The standard reference cut-off values for uPA and PAI-1 were set on 3 ng/mg of protein for uPA and 14 ng/mg of protein for PAI-1 [8].

### DNA isolation

DNA was successfully isolated from 79 fresh frozen tumor samples through protein precipitation at high salt concentration. Initial cells disruption and digestion with SDS–proteinase K, followed by the addition of high concentrations of salts (6 M sodium chloride) was done. After the proteins were discarded, DNA was extracted and precipitated with ethanol. DNA quantity and quality was measured by BioSpec-nano spectrophotometer (Shimadzu).

### PCR-RFLP analysis

An insertion/deletion polymorphism at the -675 bp position of the promoter region of *PAI-1* gene (− 675 4G/5G) was detected using the following primers: forward 5’-CACAGAGAGAGTCTGGCCACGT-3′ and reverse 5’-CCAACAGAGGACTCTTGGTCT -3’resulting in the 98 bp PCR product for 4G allele and 99 bp PCR product for 5G allele. Thermal cycling conditions were as follows: initial denaturation step at 94 °C for 5 min, 40 cycles of: 94 °C 30 s, 56 °C 30 s, 72 °C 30 s and the final elongation for 10 min at 72 °C. PCR products were visualized by electrophoresis on 2% agarose gel and digested with BslI (Thermo Scientific, Waltham, MA, USA) fast digest restriction enzyme according to manufacturer’s instructions. The digestion fragments were separated in 8% polyacrylamide gel. PAI-1 (− 675) 5G allele showed 77-bp fragment, and PAI-1 (− 675) 4G allele showed 98-bp fragment.

### Statistical analysis

For normal distribution data testing, the Kolmogorov-Smirnov and Shapiro-Wilk tests were used. Descriptive methods (frequencies, percent, mean, median, standard deviation (SD) and range) were used to summarize the data. The statistical significance level was set at *p* < 0.05 and Bonferroni correction was used for multiple testing at the same data set. For comparison of disease and treatment characteristics among different risk subgroups the Kruskal-Wallis, Wilcoxon rank sum, Pearson chi-square and Fisher exact tests were used. Methods of survival analysis (Kaplan-Meier product-limit method; median with corresponding 95% CI; log-rank test) were used for DFS, OS, EFS. The Receiver Operating Characteristics curve (ROC) methods (AUC ROC-Area Under the ROC curve according DeLong’s method; Likelihood ratio test for AUC ROC; the best cut-off value for uPA and PAI-1 was set as value with maximum sensitivity and specificity) were applied to investigate uPA and PAI-1 discriminative potential for presence/absence of relevant events (i.e. loco-regional recurrence and/or distant metastases and/or second primary of contralateral breast and/or non-breast primary cancer and/or death from any cause). The statistical analysis was done with the program R (version 3.3.2 (2016-10-31) -- “Sincere Pumpkin Patch”; Copyright (C) 2016 The R Foundation for Statistical Computing; Platform: x86_64-w64-mingw32/× 64 (64-bit); downloaded: January 21, 2017).

## Results

### Patients, disease and therapy characteristics

Fifty-six percent of patients were stage 2, 88% had tumors sized up to 30 mm, and 83% had grade 2 breast cancers. The proliferative index Ki67 was determined in 2/3 of patients with a median of 25% (range 3–90%). Fifty percent of patients had tumors with uPA tumor tissue level higher than 3 ng/mg of protein and 60% had PAI-1 higher than 14 ng/mg of protein. Significantly higher number of patients in our patients’ group had tumors with both markers either low (37.04%) or high (46.91%) compared to patients with tumors containing combination of these markers (13.58% who had low uPA and high PAI-1 and 2.47% who had high uPA and low PAI-1), (Fisher exact test, *p* < 0.0001). Patient and disease characteristics are shown in Table 1.

**Table 1:** Patient and disease characteristics

Characteristic	N (%)	Characteristic	N (%)
*Age (years)*		*ER/PR status*	81 (100)
Mean (SD)	65.6 (10.58)	ER-positive	80 (98.77)
Median (range)	66 (36–82)	ER-negative	1 (1.23)
		PR-positive	51 (62.96)
*Stage at diagnosis*		PR-negative	4 (4.94)
Stage 1	36 (44.44)	PR-unknown	26 (32.10)
Stage 2	45 (55.56)		
		*HER2 status*	
		HER2-positive	–
*Histology*		HER2-negative	81 (100)
Ductal invasive	36 (44.44)		
Lobular invasive	36 (44.44)	*Ki67 status (%)*	
Others	9 (11.12)	Mean (SD)	26.76 (20.44)
		Median (range)	25 (3–90)
*Tumor size*		Ki67 < 15%	22 (27.16)
≤ 20 mm	34 (41.98)	Ki67 ≥ 15%	29 (35.80)
20–30 mm	37 (45.68)	Ki67 unknown	30 (37.04)
> 30 mm	9 (11.11)		
Unknown	1 (1.23)	*uPA (ng/mg of protein)*	
		Mean (SD)	4.37 (3.9)
*Tumor grade*		Median (range)	3 (0.1–18.7)
Grade 1	11 (13.58)	uPA ≤ 3	41 (50.62)
Grade 2	67 (82.72)	uPA > 3	40 (49.38)
Grade 3	3 (3.70)		
		*PAI-1 (ng/mg of protein)*	
*Nodal status*		Mean (SD)	21.47 (18.89)
Node-negative	75 (92.59)	Median (range)	16 (0–140)
Node-positive	–	PAI-1 ≤ 14	32 (39.51)
Unknown^a^	5 (6.17)	PAI-1 > 14	49 (60.49)
		*uPA and PAI-1 combinations*	
		uPA ≤ 3 and PAI-1 ≤ 14	30 (37.04)
		uPA ≤ 3 and PAI-1 > 14	11 (13.58)
		uPA > 3 and PAI-1 ≤ 14	2 (2.47)
		uPA > 3 and PAI-1 > 14	38 (46.91)

^a^All clinically node-negative

All patients were treated with adjuvant endocrine therapy, among whom almost 90% received tamoxifen (TAM) while about 10% of them took aromatase inhibitors (AI). Therapy characteristics and disease outcome are shown in Table 2.

**Table 2:** Therapy characteristics and disease outcome

Characteristic	N (%)	Characteristic	N (%)
*Type of breast surgery*		*Disease relapse*	
Radical mastectomy	36 (44.44)	Yes	10 (12.35)
Breast conserving surgery	41 (50.62)	No	71 (87.65)
Other	4 (4.94)		
		*First relapse site*	
		Locoregional relapse	4 (4.94)
*Adjuvant systemic therapy*		Lung	3 (3.7)
Yes	81 (100)	Liver	1 (1.23)
No	–	Bones	4 (4.94)
		Brain	1 (1.23)
		Contralateral BC	1 (1.23)
*Adjuvant hormonal therapy*	81 (100)	Total	10 (12.35)
TAM	70 (86.42)		
TAM + GOSERELIN	2 (2.47)	*Patients’ outcome*	
AI	9 (11.11)	Dead	7 (8.64)
		Alive	74 (91.36)
*Adjuvant RT*		*Relevant events* ^a^	
Yes	45 (55.56)	Yes	15 (18.52)
No	36 (44.44)	No	66 (81.48)

^a^Relevant events: development of loco-regional recurrence and/or distant metastases and/or contralateral breast cancer and/or non-breast primary cancer and/or death from any cause

**Fig. 1:**
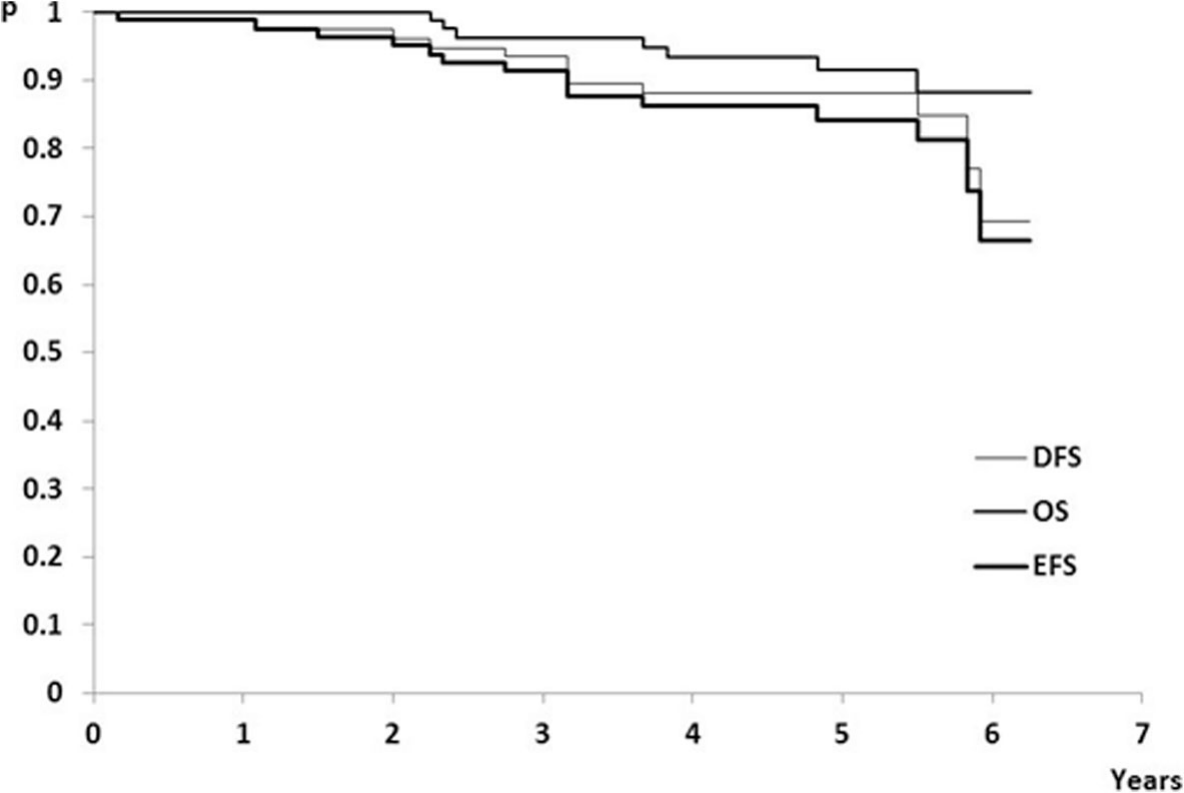
Disease-free survival (DFS), overall survival (OS), and event-free survival (EFS) in analyzed early breast cancer patients

During median follow-up period of 62 months (range: 27–75 months) disease relapse experienced 10/81 (12.35%) patients, 7/81 (8.64%) died (Table 2), while 5/7 (71.43%) patients died without evidence of breast cancer relapse. In summary, 15/81 (18.52%) patients experienced relevant events related to disease outcome. However, the median times for DFS, OS and EFS were not reached (Fig. 1).

### The association between classical prognostic factors and uPA and PAI-1 tumor tissue levels

To examine the possible association between uPA and PAI-1 tumor tissue levels with classical prognostic factors within a group with favorable prognosis we chose tumor size (less or equal to 20 mm vs. greater than 20 mm but less or equal to 30 mm vs. greater than 30 mm), tumor grade (grade 1 vs. grade 2/3), ER level (higher of equal to 50% vs. ER less than 50% of positive cells), and Ki67 level (less than 15% vs. higher or equal to 15% of positive cells). A significantly higher number of patients with tumors with Ki67 higher and equal to 15% had also uPA higher than 3 ng/mg of protein compared to patients with tumors with Ki67 less than 15% (Pearson χ2 Test, *p* = 0.039). Tumors with Ki67 higher and equal to 15% had significantly higher PAI-1 tumor tissue level compared to tumors with Ki67 less than 15% of positive cells (Wilcoxon Rank Sum Test, *p* = 0.038). The uPA and PAI-1 tumor tissue levels, tumor size, tumor grade, ER, and Ki67 are shown in Table 3.

**Table 3:** Classical prognostic factors and uPA and PAI-1 tumor tissue levels using standard reference cut-off values

Classical prognostic factors	uPA (ng/mg of protein)					PAI-1 (ng/mg of protein)				
	Values		Categories			Values		Categories		
	Mean (SD) Median (Range)	Wilcoxon rank sum	≤ 3	> 3	Pearson χ2 test	Mean (SD) Median (Range)	Wilcoxon rank sum	≤ 14	> 14	Pearson χ2 test
*Tumor size*										
≤ 20 mm	3.9 (4.3) 2.5 (0.1–18.7)	ns^a^	21 (51.2%)	13 (32.5%)	ns^b^	17.7 (14.1) 12.5 (0.0–51.8)	ns^a^	18 (56.3%)	16 (32.7%)	ns^b^
20–30 mm	4.9 (3.8) 4.0 (0.5–15.5)		15 (36.6%)	22 (55.0%)		24.6 (22.9) 17 (7.5–140)		10 (31.3%)	27 (55.1%)	
> 30 mm	4.3 (2.4) 5.1 (0.6–7.1)		4 (9.8%)	5 (12.5%)		24.6 (16.2) 24.5 (6.0–51.0)		3 (9.4%)	6 (12.2%)	
Unknown	–		1 (2.4%)	–		–		1 (3.1%)	–	
*Tumor grade*										
Grade 1	4.1 (3.1) 5.2 (0.2–8.9)	ns	5 (12.2%)	6 (15.0%)	ns	20.4 (15.3) 18.6 (0.9–46.0)	ns	5 (15.6%)	6 (12.2%)	ns
Grade 2/3	4.4 (4.0) 3 (0.1–18.7)		36 (87.8%)	34 (85.0%)		21.6 (19.5) 16.0 (0.0–140)		27 (84.4%)	43 (87.8%)	
*Estrogen receptor*										
≥ 50%	4.1 (3.6) 2.9 (0.2–15.5)	ns	20 (48.8%)	18 (45.0%)	ns	21.9 (23.0) 15.3 (0.9–140)	ns	15 (46.9%)	23 (46.9%)	ns
< 50%	4.6 (5.2) 3.3 (0.1–18.7)		21 (51.2%)	22 (55.0%)		21.1 (14.6) 16.8 (0.0–56.8)		17 (53.1%)	26 (53.1%)	
*Ki67 index*										
< 15%	3.8 (4.0) 1.8 (0.2–13.3)	ns	14 (34.2%)	8 (20.0%)	0.039	17.0 (13.0) 13.8 (0.9–56.8)	0.038	11 (34.4%)	11 (22.5%)	ns
≥ 15%	4.8 (3.4) 5.1 (0.5–15.5)		10 (24.4%)	19 (47.5%)		27.5 (25.3) 20.0 (3.0–140)		7 (21.9%)	22 (44.9%)	
Unknown	–	–	17 (41.5%)	13 (32.5%)		–		14 (43.8%)	16 (32.7%)	
Total	81 (100%)	–	41 (100%)	40 (100%)	–	81 (100%)	–	32 (100%)	49 (100%)	–

^a^Kruskal Wallis Test; ^b^Fisher exact test; *ns*-not statistically significant

### Classical prognostic factors, uPA and PAI-1 tumor tissue levels and disease outcomes (DFS, OS, EFS)

Our analysis of pairs of tumor size categories showed that patients with tumors greater than 30 mm had significantly decreased DFS and EFS compared with patients with tumors less or equal to 20 mm (Log-rank test with Bonferroni correction; *p* = 0.0056 < 0.0167 = 0.05/3) (Table 4).

**Table 4:** The effects of classical prognostic factors on DFS, OS and EFS

Characteristics	DFS (months)		OS (months)		EFS (months)	
	Median (95% CI)	Log-rank test	Median (95% CI)	Log-rank test	Median (95% CI)	Log-rank test
*Tumor size*						
≤ 20 mm	NR		NR	ns	NR	
> 20 - ≤ 30 mm	NR	0.0082	NR		71 (> 71)	0.013
> 30 mm	70 (> 38)		NR		70 (> 38)	
*Tumor grade*						
Grade 1	NR	ns	NR	ns	NR	ns
Grade 2/3	NR		NR		NR	
*Estrogen receptor*						
≥ 50%	NR	ns	NR	ns	NR	ns
< 50%	NR		70 (> 70)		70 (> 70)	
*Ki67 index*						
< 15%	NR	ns	NR	ns	NR	ns
≥ 15%	NR		NR		NR	
*uPA and PAI-1 levels*						
Both elevated	NR (> 70)		NR		NR (> 70)	ns
One elevated	NR	ns	NR	ns	NR	
Both normal	NR (> 71)		NR		NR (> 71)	

*NR-not reached; ns*-not statistically significant

**Fig. 2:**
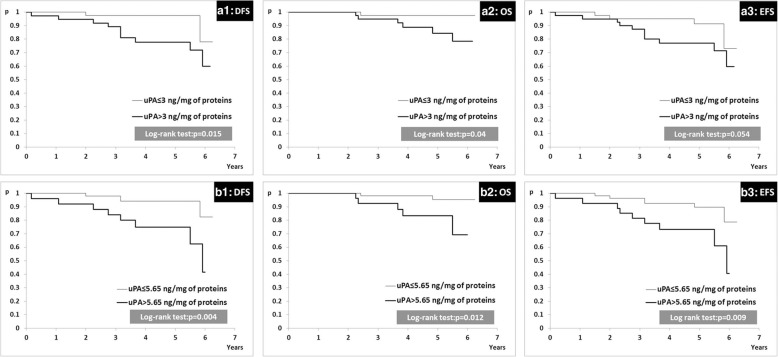
Kaplan-Meier plots for DFS, OS, EFS according to cut-off levels of uPA: (**a**) for validated reference cut-off value set at 3 ng/mg of protein; (**b**) for ROC cut-off value set at 5.65 ng/mg of protein

**Fig. 3:**
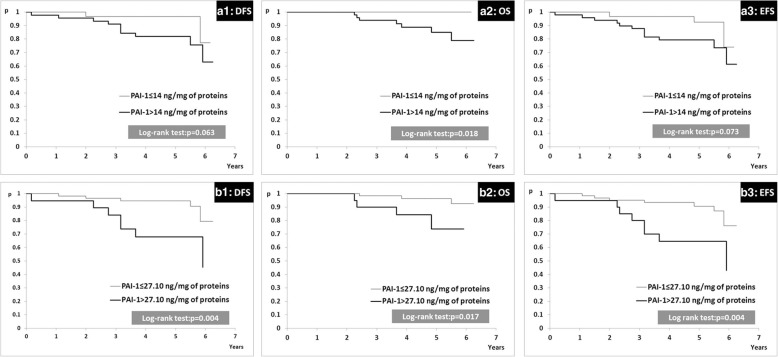
Kaplan-Meier plots for DFS, OS, EFS in relation to cut-off levels of PAI-1; (**a**) for validated reference cut-off level set at 14 ng/mg of protein, (**b**) for ROC cut-off level set at 27.10 ng/mg of protein

Although median times to events for DFS/OS/EFS were not reached, patients with uPA tumor levels higher than 3 ng/mg of protein had significantly decreased DFS and OS (Fig. 2; cases A1 and A2, respectively). In addition, patients with PAI-1 tumor tissue levels higher than 14 ng/mg of protein had significantly decreased OS (Fig.3; case A2). To test if aggregated contents of uPA and PAI-1 tumor levels might have influenced disease outcome, we divided the study group into three subgroups: subgroup 1 (*N* = 38, 46.91%), uPA and PAI-1 both elevated, subgroup 2 (*N* = 13, 16.05%), uPA elevated and PAI-1 within normal range or uPA within normal range and PAI-1 elevated, and subgroup 3 (*N* = 30, 37.04%), uPA and PAI-1 both within normal limits (Table 1). There were no differences in EFS, DFS and OS between the three subgroups (Table 4).

### The association between uPA and PAI-1 tumor tissue levels and the occurrence of the relevant events

To further evaluate the clinical significance of altered tumor levels of uPA and PAI-1 in early breast cancer, we examined the association between uPA and PAI-1 tumor tissue levels and the occurrence of the relevant events. As it could be seen in Table 5, the tumors of patients who experienced any relevant event had significantly higher values of uPA and PAI-1 in comparison with tumors of patients without such an event.

**Table 5:** uPA and PAI-1 levels in relation to the occurrence of relevant events

Biomarker	Relevant event		
	Yes	No	Wilcoxon rank sum test
*uPA values*			
Mean (SD)	6.5 (4.8)	3.9 (3.5)	*p* = 0.042
Median (Range)	5.7 (0.5–18.7)	2.7 (0.1–15.5)	
*PAI-1 values*			
Mean (SD)	33.7 (32.7)	18.7 (12.9)	*p* = 0.037
Median (Range)	27.2 (8–140)	15.1 (0–56.8)	

**Fig. 4:**
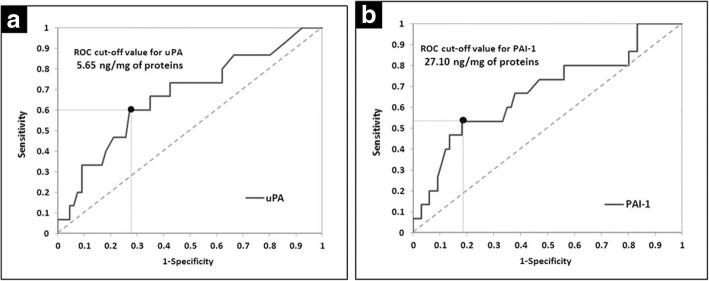
(**a**) ROC curve for the uPA; (**b**) ROC curve for the PAI-1

By applying ROC analysis, we confirmed the discriminative potential of uPA and PAI-1 for the presence/absence of relevant events with new ROC cut-off values: 5.65 ng/mg of protein for uPA and 27.10 ng/mg of protein for PAI-1 (Table 6; Fig. 4).

**Table 6:** Results of the ROC analysis for uPA and PAI-1 and relevant events

Characteristics	uPA	PAI-1
AUC ROC^a^ (95% CI)	66.9% (50.9–82.9%)	67.32% (50.8–83.8%)
Likelihood ratio test^b^	***p*** **= 0.0268**	***p*** **= 0.0099**
ROC-cut-off value^c^	5.65	27.10
Sensitivity (95% CI)	60.0% (33.3–86.7%)	53.44% (26.7–80.0%)
Specificity (95% CI)	72.7% (62.1–83.3%)	81.82% (72.7–90.9%)

^a^Area Under the ROC curve (DeLong’s method); ^b^Likelihood ratio test for AUC ROC; ^c^Value (ng/mg of protein) with maximum sensitivity and specificity

### ROC cut-off values for uPA and PAI-1 and disease outcomes (DFS, OS, EFS)

Next, we examined if there were differences in disease outcomes between subgroup of patients divided according to ROC cut-off values for uPA and PAI-1 tumor tissue levels. This analysis revealed that patients with tumors containing uPA lower or equal to 5.65 ng/mg of protein had significantly increased DFS, OS and EFS compared to patients with tumors containing uPA higher than 5.65 ng/mg of protein (Fig. 2, cases B1, B2 and B3, respectively). Similarly, patients with tumors containing PAI-1 lower or equal to 27.10 ng/mg of protein had significantly increased DFS, OS and EFS compared to patients with tumors containing PAI-1 higher than 27.10 ng/mg of protein (Fig. 3; cases B1, B2 and B3, respectively).

### -675 4G/5G *PAI-1* genotype and disease outcome

**Fig. 5:**
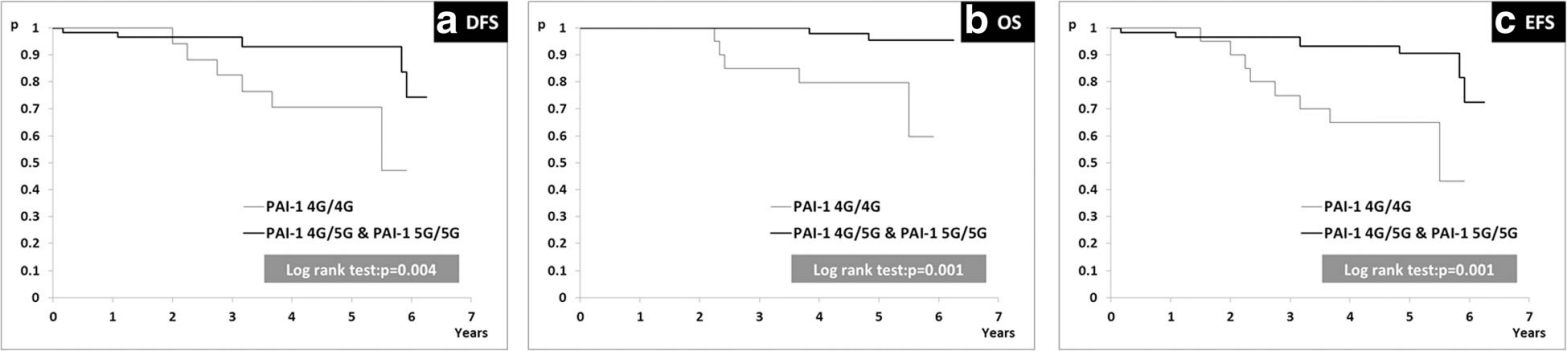
Kaplan-Meier plot of survival curves for subgroup of patients with recessive homozygote genotype (*PAI-1* 4G/4G) and subgroups of patients with heterozygote/dominant homozygote genotypes (*PAI-1* 4G/5G and *PAI-1* 5G/5G); (**a**) DFS, (**b**) OS, (**c**) EFS

The influence of − 675 4G/5G *PAI-1* genotypes on DFS, OS and EFS was estimated by Log-Rank test (Fig. 5). We observed that patients whose tumors harbor recessive genotype of insertion/deletion polymorphism at the -675 bp position of the promoter region of *PAI-1* gene (*−* 675 4G/4G) had significantly decreased DFS, OS and EFS in comparison with patients who had heterozygote (*−* 675 4G/5G) or dominant homozygote genotypes (*−* 675 5G/5G) (Log-rank test; *p* < 0.01 for DFS, OS, EFS). Furthermore, tumors with (*−* 675 4G/4G) had significantly higher tumor tissue levels of uPA than those having dominant allele in their genotype (− 675 4G/5G and − 675 5G/5G) and trend toward higher concentrations of PAI-1 (Table 7).

**Table 7:** uPA and PAI-1 levels in relation to −675 4G/5G *PAI-1* genotypes

*-*675 4G/5G *PAI-1* genotype	uPA and PAI-1 and Wilcoxon rank sum test results			
	uPA values	*p*-value	PAI-1 values	*p*-value
4G/4G		**0.032**		0.076
Mean (SD)	6.21 (4.74)		30.5 (29.76)	
Median (Rang)	5.5 (0.5–18.7)		19.8 (6.5–140)	
4G/5G and 5G/5G				
Mean (SD)	3.67 (3.21)		18.76 (12.59)	
Median (Rang)	2.7 (0.2–15.5)		15.1 (0.9–56.8)	

## Discussion

Extracellular matrix-degrading protease uPA and its inhibitor PAI-1, which promote invasion and metastasis of malignant tumors, are considered as strong prognostic factors in early stage, node-negative breast cancer [4, 26–28]. Patients with lymph node-negative breast cancer who have low intratumor uPA and PAI-1 concentrations have better disease prognosis when compared with patients who have high intratumor concentrations of uPA and/or PAI-1, which reflects tumor aggressiveness [4, 26–28].

In our single-institution retrospective study we evaluated the influence of classical prognostic factors and uPA and PAI-1 concentrations in breast cancer tissue on disease outcomes in HR-positive/HER2-negative node-negative breast cancer patients treated with adjuvant endocrine therapy. In this analysis, besides uPA and PAI-1 tumor concentrations, only tumor size was shown as prognostic factor in these women. Patients with tumors greater than 30 mm had significantly decreased DFS and EFS compared with patients with tumors less or equal to 20 mm. This is in line with the results of Dovnik and Takac [29]. Although tumors with Ki67 higher than 15% had significantly higher PAI-1 tumor tissue level compared to tumors with Ki67 less or equal to 15%, there was no difference in disease outcome between patients with lower and higher Ki67 proliferative indices.

The results of our study point to important prognostic value of uPA and PAI-1 tumor concentrations in early luminal breast cancer patients treated with adjuvant endocrine therapy. Patients with uPA higher than 3 ng/mg of protein had shorter DFS and OS, while patients with PAI-1 higher than 14 ng/mg of protein had decreased OS, which is in accordance with the results of other authors [3–8]. However, we failed to confirm that risk group discrimination is better when used the combination of both factors compared to either factors alone, probably due to low number of patients and events.

We also found that high uPA concentrations in breast cancers were associated with high PAI-1 concentrations. Among 40 patients with uPA tumor concentrations higher than 3 ng/mg of protein, 38 patients also had PAI-1 tumor concentrations higher than 14 ng/mg of protein. This is in accordance with studies of De Cremoux et al. [26] and Lampelj et al. [30] that reported significant positive correlation between uPA and PAI-1 tumor levels. However, in our study a remarkably higher frequency of patients (47%) had higher tumor tissue levels of both uPA and PAI-1 when compared with data obtained by Lampelj et al., where 21% of patients with no axillary lymph node involvement had high levels of both biomarkers [30]. The observed dissimilarity in frequencies of breast cancer patients with high levels of uPA and PAI-1 between our study and study by Lampelj et al. might be attributed at least in part to differences in a cohort selection.

Furthermore, in comparison with standard reference cut-off values for uPA and PAI-1 (3 ng/mg of protein for uPA and 14 ng/mg of protein for PAI-1) the ROC cut-off values (5.65 ng/mg of protein for uPA and 27.10 ng/mg of protein for PAI-1) seemed to have more reliable discriminative potential to separate patients into subgroups with better and poorer disease outcome. Our finding suggests that increase of standard uPA and PAI-1 cut-off values may contribute to more powerful prognostic impact of these biomarkers in the examined group of patients. Moreover, these patients would highly benefit from this analysis in terms of further classification in groups with better and worse prognosis. Nevertheless, we are also aware that low number of the examined patients and follow-up period of 62 months represent limitations of the present research. Despite these limitations, homogeneity of the investigated group of breast cancer patients certainly represents the strength of our study.

We did not perform the immunohistochemical analysis of uPA and PAI-1 tumor and lymph node expression levels, nor did we do the analysis of the influence of these parameters on disease outcome. The recently published study showed that there was no influence neither of uPA, nor PAI-1 protein expression levels on disease outcome [31]. The correlation between ELISA and IHC determination of uPA and PAI-1 and significance of their influence on disease outcome of breast cancer are not consistent [5–10, 12]. Therefore, for now ELISA remains the gold standard for determining uPA and PAI-1 tumor concentrations. Although IHC method using formalin -fixed paraffin- embedded tumor tissue would have been more acceptable for routine clinical practice, IHC determination of uPA and PAI-1 is not currently recommended for use in clinical practice [32]. However, the establishing, validation and standardization a method for measuring uPA and PAI-1 using formalin fixed paraffin embedded tumor tissue should be a subject of future research [32].

Previously reported studies aiming to investigate the influence of genetic variability on PAI-1 synthesis reported that the 4G allele of *PAI-1* promoter region is associated with higher levels of PAI-1 in serum or in tumor tissues [20, 33]. However, this was not confirmed in other published papers and the results remain inconsistent [34, 35]. Our study did not demonstrate statistically significant association between *PAI-1* promoter region polymorphism and PAI-1 tumor concentrations in analyzed breast cancers. However, a trend towards higher PAI-1 tumor concentrations in tumors harboring recessive homozygote genotype of *PAI-1* (*−* 675 4G/4G) (Table 7) reported in our study, indicates that expanding the size of the investigated group might influence this result.

We showed significant correlation between recessive homozygote genotype (− 675 4G/4G) of *PAI-1* and higher uPA tumor tissue level. This was not expected since 4G allele was shown to be associated with higher PAI-1 expression and consequently, lower uPA levels [21]. There are a couple of possible explanations for this discrepancy. Even though recessive allele leads to higher expression of the PAI-1 protein, post-translational modification and interaction with other proteins might lead to its decreased activity that might influence its inhibitory effect on uPA. Another explanation might be the existence of other mechanisms that affect uPA levels and are dominant comparing to the inhibition related to PAI-1 activity.

We further evaluated the possible prognostic value of − 675 4G/5G *PAI-1* gene variants. This analysis showed that subgroup of patients with heterozygote/dominant homozygote genotypes (− 675 4G/5G and − 675 5G/5G) had significantly longer DFS, OS and EFS when compared with subgroup of patients with recessive homozygote genotype (− 675 4G/4G). These results show the importance of *PAI-1* gene polymorphism as a prognostic biomarker in luminal HER2-negative node-negative breast cancer patients. Our data are in line with the results of Zhang et al. who reported that breast cancer patients homozygous for the 4G allele of *PAI-1* gene had significantly decreased DFS and OS in comparison with patients homozygous for the 5G allele [36]. In contrast to these results, the study of Lei et al. showed that 5G/5G homozygous breast cancer patients had lower OS in comparison with patients with 4G/4G and 4G/5G *PAI-1* genotypes [37]. These differences might be explained by specific demographic and molecular factors which can change PAI-1 effects [38].

Our analysis confirmed the usefulness of uPA and PAI-1 biomarkers for assessing prognosis in patients with node-negative, HR-positive/HER2-negative early breast cancers treated with adjuvant endocrine therapy. Measurements of uPA and PAI-1 concentrations in breast cancer tissue extracts by FEMTELLE® ELISA, may still maintain their role as an important part of the individualized therapy decision making.

## Conclusions

Our study showed that both uPA and PAI-1 tumor tissue levels may have an influence on disease outcome in node-negative, HR-positive/HER2-negative breast cancer patients treated with adjuvant endocrine therapy. In addition, our ROC cut-off values for both protein markers might better separate patients with poorer prognosis. Results of our study suggest that subgroup of patients with recessive homozygote genotype of *PAI-1* (*−* 675 4G/4G) might have worse disease outcome when compared with patients with heterozygote/dominant homozygote genotypes (*−* 675 4G/5G and *−* 675 5G/5G).

## Abbreviations

AI: Aromatase inhibitors.
AUC ROC: Area Under the ROC curve
DFS: Disease-free survival
EFS: Event-free survival
ER: Estrogen receptor
HR: Hormone receptor
IHC: Immunohistochemistry
OS: Overall survival
PAI-1: Plasminogen activator inhibitor type 1
PR: Progesterone receptor
ROC: Receiver Operating Characteristics
SD: Standard deviation
TAM: Tamoxifen
TBS: Tris Buffered Saline
uPA: Urokinase-type plasminogen activator
